# Isoindolinone-Derived PET Tracers for Molecular Imaging of mHTT Aggregates in Huntington’s Disease

**DOI:** 10.1021/acsmedchemlett.5c00557

**Published:** 2025-10-07

**Authors:** Yinlong Li, Lu Wang, Zhong Pei, Stewart A. Factor, Steven H. Liang

**Affiliations:** † Department of Radiology and Imaging Sciences, 1371 Emory University, 1364 Clifton Road, Atlanta, Georgia 30322, United States; ‡ Center of Cyclotron and PET Radiopharmaceuticals, Department of Nuclear Medicine and Key Laboratory of Basic and Translational Research on Radiopharmaceuticals, The First Affiliated Hospital of Jinan University, Guangzhou 510630, Guangdong, China; § Department of Neurology, The First Affiliated Hospital, Sun Yat-sen University, Guangzhou 510080, China; ∥ Jean and Paul Amos Parkinson’s Disease and Movement Disorder Program, Department of Neurology, Emory University School of Medicine, Atlanta, Georgia 30322, United States

**Keywords:** Huntington’s disease, mutant huntingtin, structure−activity relationship, positron emission tomography, radiotracers

## Abstract

Mutant huntingtin (mHTT) aggregates represent a key pharmacodynamic biomarker of Huntington’s disease (HD). The development of positron emission tomography (PET) tracers targeting mHTT addresses a critical unmet need by enabling the noninvasive quantification of pathological burden *in vivo*. The first-generation tracer, [^11^C]­CHDI-180R, a benzoxazole derivative, laid the foundation for this effort. Subsequent analogs such as [^11^C]­CHDI-626 and [^18^F]­CHDI-650 were developed to improve *in vivo* performance; however, key challenges including limited metabolic stability and suboptimal selectivity persisted. To address these limitations, a recent study introduced a new class of isoindolinone-derived candidate tracers, including [^11^C]­CHDI-009, [^18^F]­CHDI-385, and [^18^F]­CHDI-386, identified through systematic structure–activity relationship (SAR) optimization. These next-generation tracers exhibit markedly enhanced binding affinity, selectivity, and translational potential, offering valuable tools to investigate mHTT pathology and its role in HD progression.

## Graphical abstract

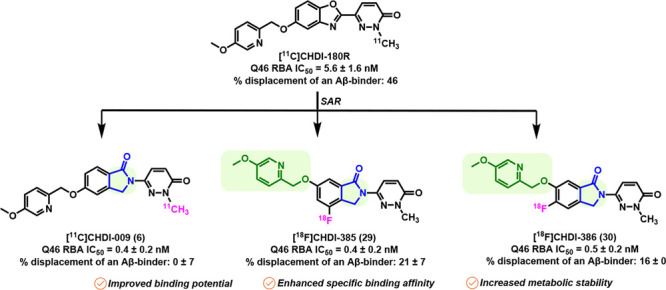

Huntington’s disease (HD) is an inherited neurodegenerative disorder characterized by progressive motor and psychiatric impairment as well as cognitive decline which strikes people in the prime of life and ultimately leads to nursing home placement and death.^1–3^ Genetically, HD is caused by an abnormal CAG trinucleotide repeat expansion in the huntingtin (HTT) gene, leading to an elongated polyglutamine (polyQ) stretch in the HTT protein.^4–6^ A repeat expansion above 39 results in 100% penetrance. A pathological hallmark of HD is the progressive accumulation of aggregated mutant huntingtin (mHTT) protein in the nucleus and cytoplasm of neurons,^7,8^ which disrupts cellular homeostasis and causes widespread neurodegeneration, most notably in the striatum (caudate nucleus) and cortex.^9–11^ Although relatively uncommon, with a prevalence of 5–10 per 100,000 individuals in many developed regions, and a greater at risk population, HD continues to pose a major challenge for therapeutic development, as no disease modifying therapy has been established to date.^12,13^ Substantial efforts have been devoted to developing HTT-lowering therapies with the prospect that they will slow disease progression.^14,15^ However, the long-term safety and efficacy of reducing mutant HTT alone or along with wild-type HTT remains unresolved and is being actively investigated.

A critical barrier to evaluating the effectiveness of lowering HTT levels is the lack of a noninvasive, quantitative method to measure mHTT in the brain.^16^ Positron emission tomography (PET), which enables *in vivo* imaging using radioligands targeted to specific molecules, holds promise for tracking neurodegenerative disease pathology.^17,18^ For HD, the development of PET tracers capable of selectively binding mHTT aggregates could enable direct assessment of disease staging and treatment effect.^19^ To address this need, the CHDI Foundation, a nonprofit organization, has spearheaded a major effort to develop PET radiotracers targeting mHTT aggregates. As a result of this initiative, several candidate tracers have been reported to date (Figure 1). The first-generation tracer [^11^C]­CHDI-180R, a benzoxazole-based compound with an IC_50_ of 5.6 ± 1.6 nM, demonstrated initial promise for imaging mHTT aggregates and was advanced to Phase 1 clinical trials.^20,21^ However, the tracer exhibited substantial nonspecific binding to pathogenic protein aggregates in Alzheimer’s disease (AD) brain homogenates, revealing its limited selectivity for mHTT. A structurally distinct analog [^11^C]­CHDI-626 retained strong binding affinity toward mHTT (IC_50_ = 3.6 ± 1.6 nM) and showed improved selectivity over amyloid-β (Aβ) and tau aggregates, but the presence of a brain-penetrant radiometabolite impeded its further clinical translation.^22,23^ Further structure–activity relationship (SAR) optimization identified a fluorine-18-labeled tracer [^18^F]­CHDI-650, which exhibited improved binding potency (IC_50_ = 1.1 ± 0.3 nM) and enhanced pharmacokinetic properties.^24^ Kaur et al. also developed an ^18^F-labeled analog of CHDI-180R ([^18^F]**1**), which showed comparable imaging performance to [^11^C]­CHDI-180R in rodents and nonhuman primates (NHPs).^25^ While these early benzoxazole-based tracers established a proof of concept for imaging mHTT *in vivo*, challenges such as off-target binding, modest binding affinity and metabolic instability hindered their translational potential. To address these limitations, Liu et al. recently reported a new class of isoindolinone-based PET tracers including [^11^C]­CHDI-009 (**6**), [^18^F]­CHDI-385 (**29**) and [^18^F]­CHDI-386 (**30**) with improved selectivity and pharmacokinetics (Figure 2).^26^

**Figure 1:**
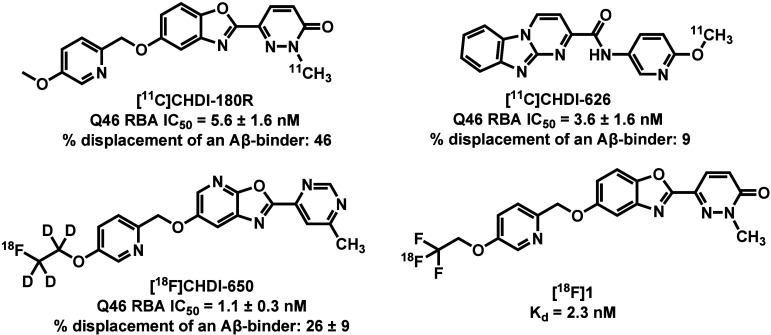
Representative mHTT-targeted PET tracers.

**Figure 2:**
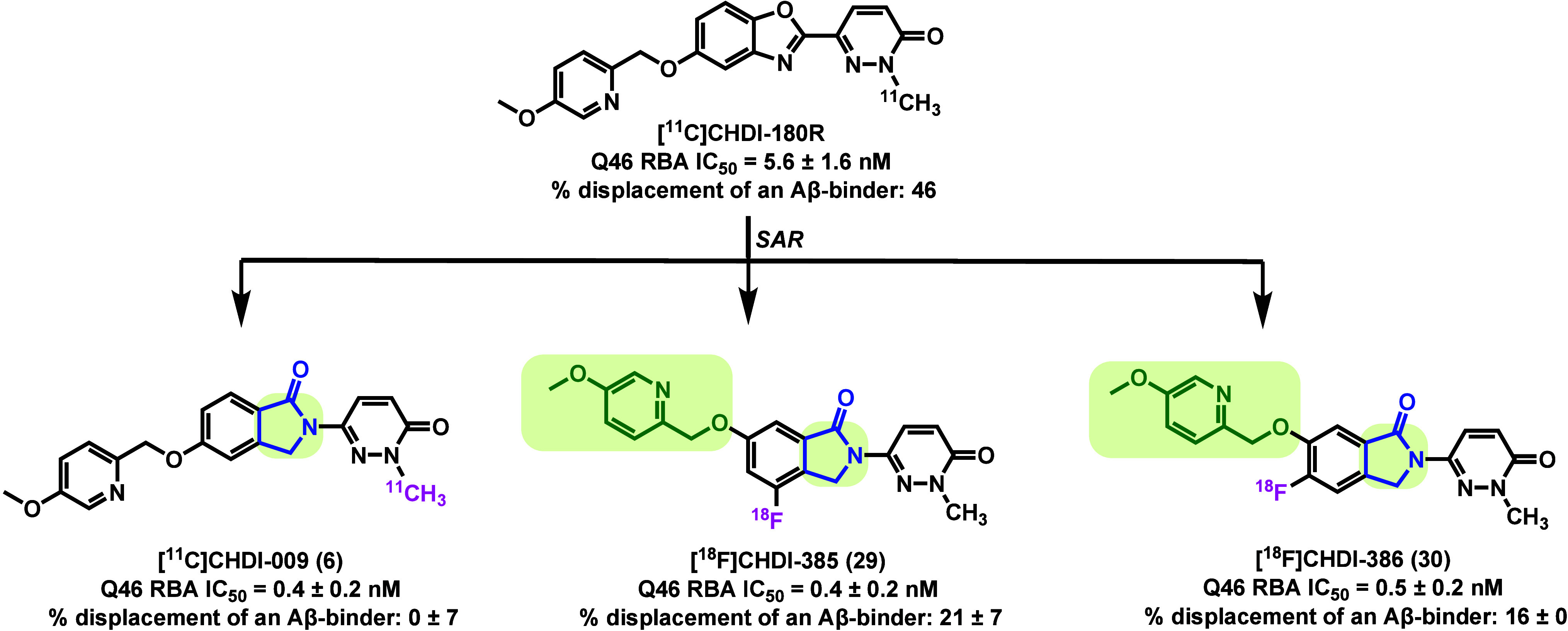
A novel series of isoindolinone-based PET tracers derived from [^11^C]­CHDI-180R.

The SAR optimization in this study commenced with systematic modifications to the bicyclic core in CHDI-180R, with the goal of assessing their impact on mHTT binding affinity and target selectivity. Binding potency (IC_50_) to mHTT was measured via radioligand competition assays using Exon1-Q46 aggregates. In parallel, off-target interactions were evaluated using AD brain homogenates (ADbhRBA) to determine displacement from Aβ and tau aggregates. This dual-screening strategy led to the identification of several isoindolinone-based analogs with markedly improved profiles: compound **6** (IC_50_ = 0.4 ± 0.2 nM), compound **29** (IC_50_ = 0.4 ± 0.2 nM), and compound **30** (IC_50_ = 0.5 ± 0.2 nM). All three exhibited high potency and minimal off-target binding (0 ± 7% to 21 ± 7% ADbh RBA) relative to CHDI-180R (Table 1). Meanwhile, all three compounds exhibited sufficient unbound fraction in brain homogenate (*f*_u,b_ ≥ 0.06) and were not identified as efflux substrates in MDR1 (P-gp) and BCRP (breast cancer resistance protein) transporter assays (efflux ratio ≤ 2.0). Since no significant pharmacophoric differences were observed between the benzoxazole and isoindolinone cores, energy minimization studies were conducted to explore potential structural explanations for the divergent selectivity profiles of compound **6** and CHDI-180R (Figure 3). The calculations revealed that the isoindolinone core in compound **6** adopts a planar conformation, comparable to that of the benzoxazole core in CHDI-180R. However, these spatial features did not correlate with their differential off-target binding to AD brain homogenates, suggesting that conformational alignment may not fully account for the observed selectivity profile.

**Table 1:** Comparison of the *In Vitro* Profile of CHDI-180R with Candidate Compounds **6**, **29**, and **30** ^a^

Compound#	Exon1-Q46 RBA IC_50_ values (nM)	*f*_u,b_	ADbh RBA (%)	MDR1 Efflux ratio	BCRP Efflux ratio
CHDI-180R	1.3 ± 1.0	0.06	51.4 ± 4	0.6	1.1
6	0.4 ± 0.2	0.10	0 ± 7	0.4	2.0
29	0.4 ± 0.2	0.06	21 ± 7	0.9	0.9
30	0.5 ± 0.2	0.1	16 ± 0	1	0.9

a Exon1-Q46 RBA IC_50_ values (nM) are reported as the geometric mean from at least two independent experiments, each performed in triplicate. ADbh RBA values represent the percentage displacement of 2 nM radioligand by 1 μM test compound and are expressed as the arithmetic mean of at least two replicates. The data was adapted from ref 26. Copyright 2025 American Chemical Society.

**Figure 3:**
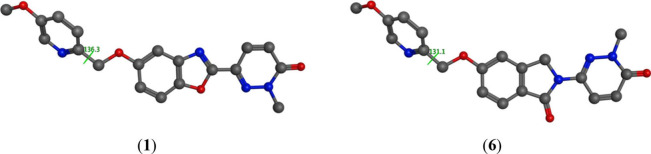
Dihedral angle analysis of compounds CHDI-180R (**1**) and **6** (−136.3° vs 131.1°). The data was adapted from ref 26. Copyright 2025 American Chemical Society.

To evaluate the *in vivo* binding specificity of compounds **6**, **29**, and **30**, the authors synthesized tritium-labeled analogs and conducted *in situ* autoradiography (ARG) studies using brain sections from homozygous zQ175 HD mouse model and wild-type (WT) controls. All three radioligands demonstrated highly specific binding in the brains of zQ175 mice, while only background-level signals observed in WT mice lacking mHTT expression (Figure 4). Co-incubation with unlabeled parent compound effectively blocked the radioligands’ uptake in zQ175 brain sections, confirming low nonspecific binding and high target selectivity for mHTT aggregates. In particular, compounds **6**, **29**, and **30** exhibited more than 2-fold higher specific binding compared to CHDI-180R, highlighting their superior *in vitro* imaging performance and translational potential.

**Figure 4:**
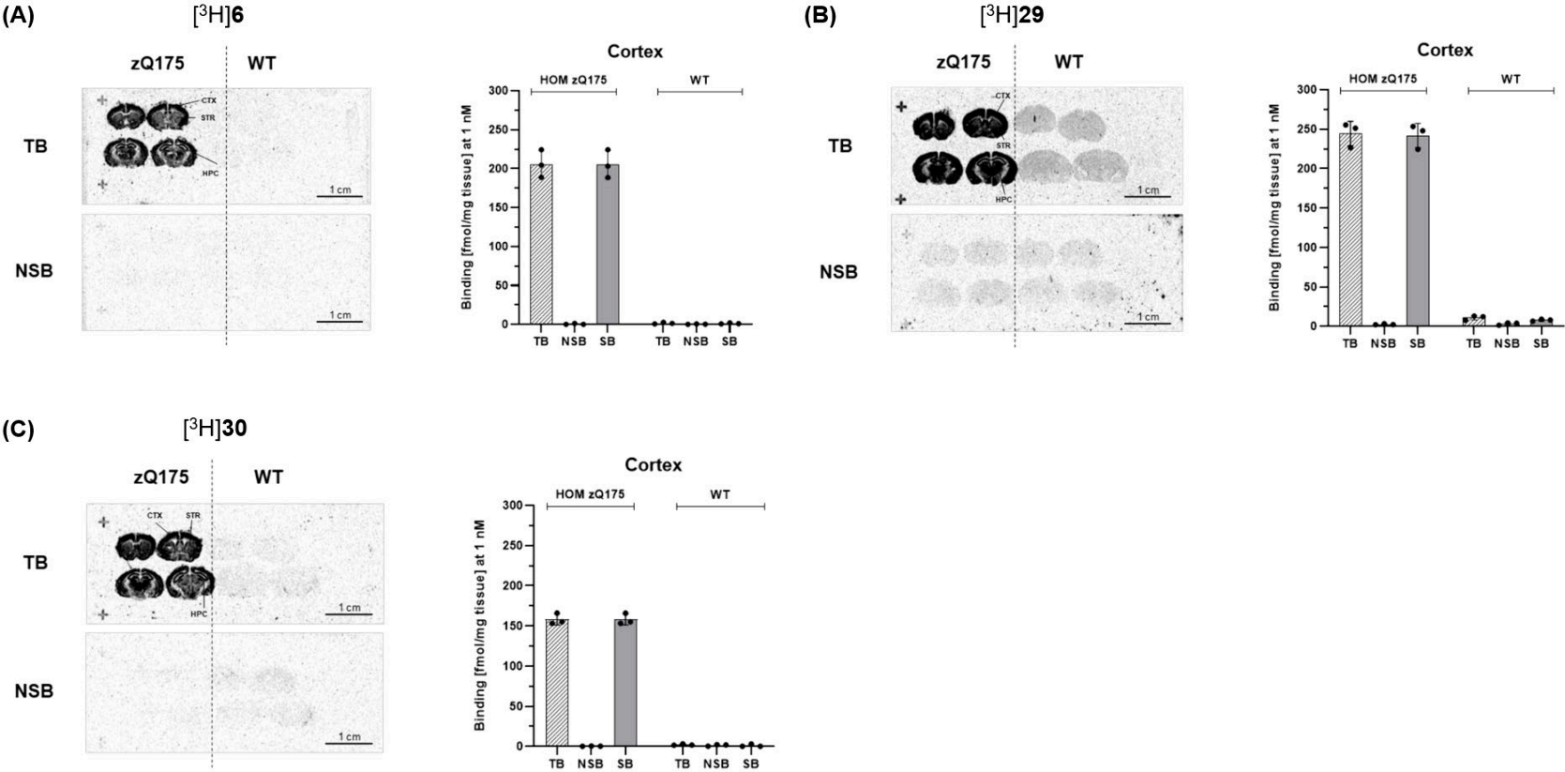
In Situ ARG studies of [^3^H]**6**, [^3^H]**29** and [^3^H]**30** in 12-month-old HOM zQ175 HD mouse and WT control brain sections. The data was adapted from ref 26. Copyright 2025 American Chemical Society.

To enable preclinical PET imaging, the authors performed radiosynthesis of the corresponding tracers. As shown in Figure 5, [^11^C]**6** was synthesized via *N*-methylation of precursor **32** using [^11^C]­MeOTf, yielding a nondecay-corrected radiochemical yield (RCY) of 12–19%. [^18^F]**29** was obtained through copper-mediated [^18^F]­fluorination of the corresponding stannane precursor **33**, with RCY of 0.6–1.3%. In contrast, radiosynthesis of [^18^F]**30** posed greater synthetic challenges and was ultimately achieved via radiofluorination of a spirocyclic iodonium ylide (SCIDY)^27–29^ precursor **34**, affording [^18^F]**30** in 4.8–7.0% RCY. Despite the modest yields, all three tracers were produced in sufficient quantities for PET imaging studies in rodents and NHPs.

**Figure 5:**
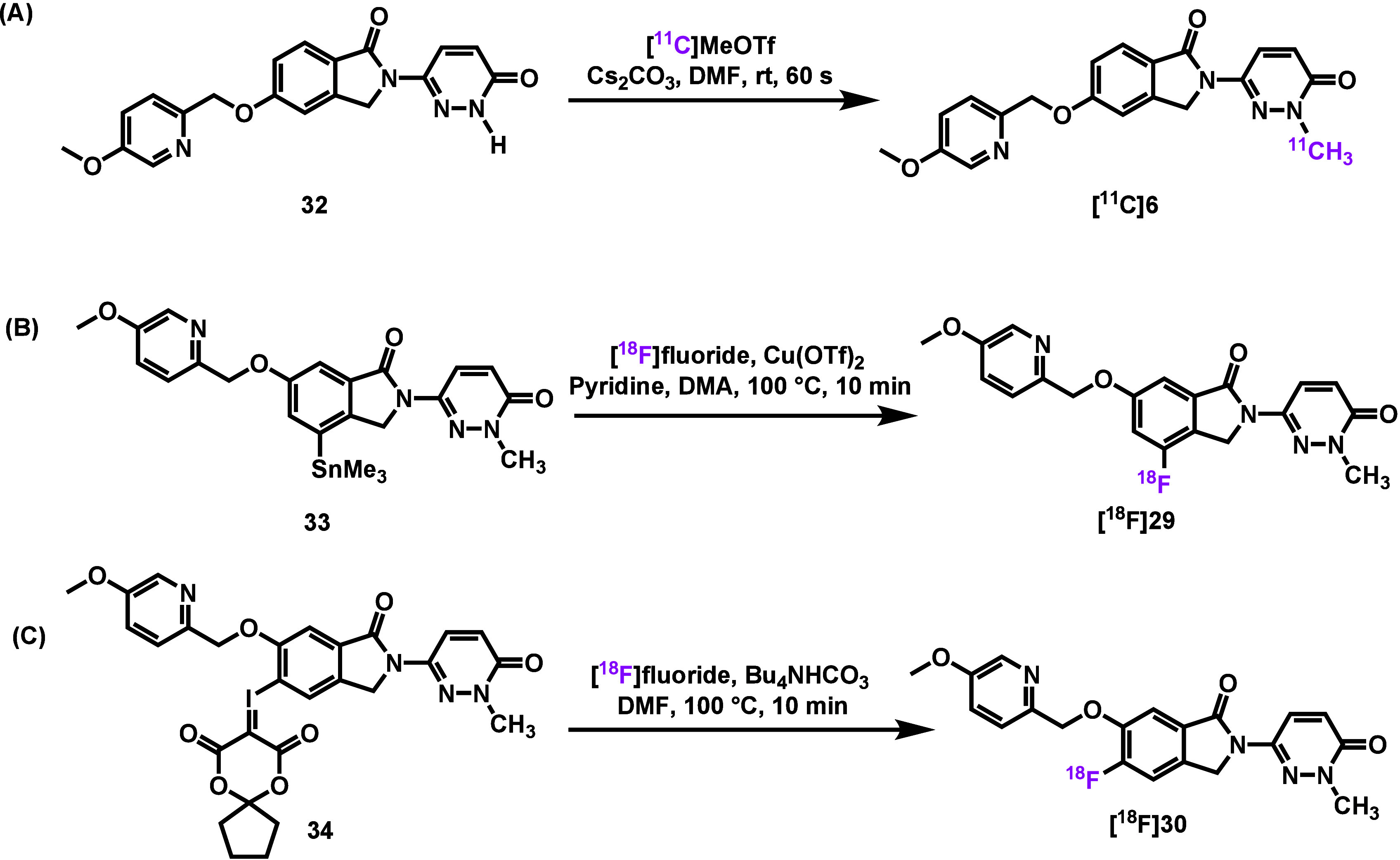
Radiosynthesis of [^11^C]**6**, [^18^F]**29** and [^18^F]**30**. The data was adapted from ref 26. Copyright 2025 American Chemical Society.

Preclinical PET imaging studies of [^11^C]**6**, [^18^F]**29** and [^18^F]**30** were conducted in heterozygous (HET) zQ175DN mice and age-matched WT controls. All three tracers demonstrated rapid brain penetration and favorable metabolic stability. Notably, tracer washout was significantly slower in HET mice compared to WT mice, likely reflecting specific binding to mHTT aggregates in the HET mice. Kinetic modeling using Logan graphical analysis with an image-derived input function (IDIF) enabled reliable estimates of tracer distribution. The volume of distribution (*V*_T_) values in HET zQ175DN mice were approximately 2-fold higher than those in WT mice, indicating specific target engagement *in vivo* (Table 2). PET imaging was also performed in healthy cynomolgus monkeys to evaluate translational potential. The mean time to reach maximum concentration (*T*_max_), mean maximum concentration (*C*_max_), and the *C*_max_/C_60 min_ ratio were generally consistent across all three tracers. A reliable *V*_T_ and the plasma-to-tissue transfer rate constant (*K*_1_) were estimated by two-tissue compartment model (2TCM), highlighting the favorable profile of these tracers for further clinical translation.

**Table 2:** Summary of PET Imaging Results for [^11^C]**6**, [^18^F]**29** and [^18^F]**30** in 9-Month-Old Male zQ175 Mice (*n* = 8) or Healthy Cynomolgus Monkeys Using a Whole Brain Volume of Interest (1 x Male, 2 x Female)^a^

	zQ175 mouse (9 month)		Cynomolgus monkey				
Ligands	VT(IDIF) HET (mL/cm^3^)	VT(IDIF) WT (mL/cm^3^)	*T*_max_ (min)	*C*_max_(g/mL)	*C*_max_/C_60 min_	*V*_T_(mL/cm^3^)	*K*_1_(mL/cm^3^/min)
[^11^C]6	0.84 ± 0.06	0.45 ± 0.04	2.92 ± 0.83	2.04 ± 0.66	9.15 ± 3.33	0.61 ± 0.16	0.26 ± 0.05
[^11^C]29	1.81 ± 0.22	0.53 ± 0.03	3.50 ± 0.00	1.43 ± 0.54	3.67 ± 1.16	0.71 ± 0.12	0.18 ± 0.02
[^11^C]30	0.79 ± 0.10	0.38 ± 0.03	3.50 ± 0.00	1.73 ± 0.09	2.16 ± 0.64	0.57 ± 0.26	0.15 ± 0.08

a Data are presented as mean ± standard deviation (SD). The data was adapted from ref 26. Copyright 2025 American Chemical Society.

## Future Outlook

Given the limited target specificity observed with the first-generation PET tracer [^11^C]­CHDI-180, the CHDI foundation initiated further SAR optimization based on the CHDI-180 scaffold. This goal of their work was to reduce off-target binding and enhance mHTT affinity. This effort led to the identification of compounds **6**, **29**, and **30** as promising lead candidates. These compounds exhibited significantly improved binding characteristics and were subsequently radiolabeled with carbon-11 and fluorine-18 to yield PET tracers for preclinical evaluation. Their binding potential was validated through both *in vitro* ARG and *in vivo* PET imaging studies, confirming high specificity, robust brain penetration, and favorable pharmacokinetics, thereby supporting their clinical translation as viable radiotracers for imaging mHTT aggregates. Despite this progress, several key challenges and opportunities remain. To date, the majority of mHTT-targeted PET ligands have been derived from benzoxazole or benzo­[4,5]­imidazo­[1,2-*a*]­pyrimidine scaffolds. Future research efforts should prioritize expanding the chemical diversity of ligand scaffolds to explore new binding modalities. While binding affinity and target selectivity have improved through years of SAR optimization, further enhancement is needed, particularly in achieving minimal cross-reactivity with Aβ and tau aggregates. Considering species differences between animal models and humans, as well as variations in clinical manifestation, the ultimate goal of this work is to validate these PET tracers in patients with HD through proof-of-concept studies. Addressing all these limitations will be essential to develop the next generation of highly selective, brain-penetrant PET tracers, which will be instrumental in monitoring disease progression, evaluating therapeutic efficacy, and ultimately accelerating the development of effective treatments for HD.

## Notes

No unexpected or unusually high safety hazards were encountered

## Abbreviations

HD: Huntington’s disease
HTT: huntingtin
polyQ: polyglutamine
mHTT: mutant huntingtin
PET: positron emission tomography
AD: Alzheimer’s disease
Aβ: amyloid-β
SAR: structure–activity relationship
NHPs: nonhuman primates
IC_50_: half maximal inhibitory concentration
ADbhRBA: Alzheimer’s disease brain homogenate radioligand binding assay
*f*_u,b_: unbound fraction in brain homogenate
P-gp: P-glycoprotein
BCRP: breast cancer resistance protein
ARG: autoradiography
WT: wild-type
HOM: homozygous
RCY: radiochemical yield
SCIDY: spirocyclic iodonium ylide
HET: heterozygous
IDIF: image-derived input function
VT: volume of distribution
*T*_max_: mean time to reach maximum concentration
*C*_max_: mean maximum concentration
2TCM: two-tissue compartment model
SD: standard deviation
